# Breaking Barriers: Stakeholder Insights into Physical Activity, Exercise, and Dietary Behaviours Among Individuals with Phenylketonuria (PKU)

**DOI:** 10.3390/healthcare14040440

**Published:** 2026-02-09

**Authors:** Annabelle G. Skidmore, Anita MacDonald, Adam J. Herbert, Kiara Lewis, Lewis A. Gough

**Affiliations:** 1 Human Performance and Health Research Group, Birmingham City University, Birmingham B15 3TN, UK; annabelle.skidmore@mail.bcu.ac.uk (A.G.S.); adam.herbert@bcu.ac.uk (A.J.H.); lewis.gough@bcu.ac.uk (L.A.G.); 2 Birmingham Children’s Hospital, Birmingham B4 6NH, UK; anita.macdonald@nhs.net

**Keywords:** Phenylketonuria, PKU, phenylalanine, physical activity, exercise, nutrition, rare condition

## Abstract

**Background/Objectives:** In Phenylketonuria (PKU), engaging in regular physical activity and exercise (PA/E) is important for physical and psychological health, but additional considerations may be required to facilitate uptake and performance as well as to optimise metabolic control. The aim of this study, therefore, was to investigate the stakeholder perspectives on the barriers, facilitators, and solutions to completing PA/E, sport, and nutrition in PKU. **Methods**: In total, 7 in-person and 6 online semi-structured focus groups (FGs) were conducted with individuals with PKU (*n* = 31), caregivers (*n* = 13), clinicians (*n* = 17), and medical industry professionals (*n* = 14) in PKU (*n* = 75 total participants). Three main questions about the barriers, facilitators, and solutions to performing PA/E with PKU were explored. Identified themes were mapped onto the capability, opportunity, motivation, and behaviour (COM-B) model of behaviour change with anonymous quotes from relevant stakeholders used to illustrate the findings. **Results**: Five common themes were identified. Most notably, individuals with PKU and their caregivers stated fatigue, poor recovery, low energy, and fear around the impact of exercise on blood phenylalanine (Phe) control were barriers to PA/E. Individuals with PKU were aware of the potential benefits of exercise, stating PA/E impacted positively on their mental well-being, daily functioning, and happiness and improved their self-confidence and long-term health. Identified solutions to PA/E participation included greater knowledge in regard to the impact of PA/E on Phe levels, improvements in advice on amount and supplementation with protein substitutes, tailored PKU nutritional advice, more awareness of PA/E within and outside the PKU community, specific PKU guidelines for PA/E, more scientific research, and PA/E events. Misalignment was evident, such that individuals with PKU reported additional barriers to PA/E, whereas other key stakeholder groups perceived the same barriers as the general public. **Conclusions**: There seems to be a misalignment between individuals with PKU, caregivers, clinicians, and industry professionals regarding PA/E, sport, and nutrition. Individuals with PKU and caregivers reported additional barriers to undertaking PA/E, sport, and nutrition compared to the general public. This suggests that further education and collaboration is needed through stakeholders to better understand how such barriers could be overcome in respect of PA/E, sport, and nutrition in individuals with PKU.

## 1. Introduction

In healthy individuals, it is generally accepted that physical activity and exercise (PA/E) can improve mood and cognition [1], decrease depressive symptoms [2], reduce obesity risk [3], and may improve bone density [4], contributing to maintenance of long-term health [5]. However, for those living with a chronic condition such as cardiovascular disease (CVD), hypertension, cancer, and type 2 diabetes (T2D) [6], participation in PA/E and sport may present further difficulties. A chronic condition presents additional barriers for participation in PA/E [7], and optimisation of exercise and nutrition to support this may pose many challenges beyond those usually experienced by the general population, such as motivation, time, and resources [8]. Research has shown individuals with physical disabilities or chronic conditions have lower physical activity levels than the general population [9,10]. Based on this evidence, it is intuitive to suggest that these individuals may potentially gain physical and mental health benefits from regular engagement in PA/E [11,12], which may subsequently reduce premature mortality and risk of additional health co-morbidities and thus improve individuals’ quality of life [13,14].

Phenylketonuria (PKU) is a rare, genetic metabolic disorder associated with a deficiency of the enzyme phenylalanine hydroxylase (PAH), where individuals with the condition have a reduced ability to convert the amino acid phenylalanine (Phe) to tyrosine (Tyr). Uncontrolled accumulation of Phe in the blood and brain causes physiological, neurological, and intellectual disabilities [15]. Several physiological and cognitive issues specific to PKU are reported [16], including increased oxidative stress [17,18], increased levels of fatigue associated with exercise [19,20], mood disturbances [21], poor bone health [22,23,24], and cognitive impairment (reduced sustained attention and processing speed) [21,25,26]. In addition, social exclusion, disordered eating, gastrointestinal (GI) issues, and poor quality of life and mental health have been reported in individuals with PKU compared with the general population [27,28,29]. Data from a cross-sectional observational study in Brazil found over 96% of adolescents with PKU (*n =* 94) were sedentary and 19% reported being overweight, which was significantly linked to body composition [30]. Additionally, data from a small-scale study conducted in the United Kingdom [31] found that both children and adults with PKU were significantly more sedentary than a control group. The authors reported that moderate physical activity duration (MPAD) in adults with PKU was 27 min per week, which is not equivalent to one day of activity stated in the European (EU) guidelines [15,32] and would class them as “inactive” according to the UK physical activity guidelines [33]. This evidence suggests that individuals with PKU are more inactive compared to generally healthy controls, which may suggest they have additional barriers to PA/E, sport, and nutrition.

The importance of sports nutrition to support exercise within PKU has been highlighted by González-Lamuño et al., [34] and described by Rocha et al., [35] via a three-patient case report within elite athletes. This resulted in recommendations including monitoring energy intake whilst maintaining a high carbohydrate diet and optimal hydration status, though this advice was based on evidence from healthy populations rather than experimental research in individuals with PKU. The total protein equivalent intake from natural protein and low Phe protein substitutes of the strength-trained individual was 1.37 g^.^kg^.^BM day^−1^ and 1.47 g^.^kg^.^BM day^−1^ for the female rugby player (England National Team) with PKU. Both intakes are well below the recommendations from sports nutrition guidelines [36]. Based on this evidence, albeit limited, those aiming for sporting performance with PKU are currently significantly below the nutritional guidelines for protein intake. This evidence suggests that athletes with PKU may have additional barriers to non-PKU athletes in respect of sporting performance, which warrants further exploration.

Considering the potential barriers to PA/E, sport, and nutrition in PKU, two primary guidelines have been produced for the EU [15] and by industry (Nutricia) [37]. Specifically, the industry guidelines are almost entirely built upon research in non-PKU cohorts, which is a concern given that due to the altered protein metabolism and that states of catabolism could increase Phe and therefore compromise long-term health. Given the unique physiology of PKU, it is important that any guideline is built upon research specifically in cohorts with PKU. In addition, the EU guidelines state, “Patients with PKU should be encouraged to participate in exercise and sporting activities and maintain adequate nutrition and energy intake to ensure optimal performance” [15]. This is a vague recommendation and is based on a low level of evidence for individuals with PKU. Future developments are required to produce appropriate guidelines to suit the needs of individuals with PKU, where stakeholder feedback is an important part of this process.

Whilst it is important to explore the barriers to PA/E, sport, and nutrition in PKU, it is also important to capture the facilitators and solutions. To work towards potential solutions, it is also important that these factors are obtained from all interested stakeholders to improve support for any subsequent recommendations. To date, this has not been considered for PKU, unlike other chronic conditions such as diabetes [38], obesity [39], and musculoskeletal disorders [40], where typically patients, clinicians, industry, and charities are consulted. Exploring these perceived barriers within PKU may prove beneficial, as research has been successfully completed in a variety of health conditions to help with PA/E prescription, including the aforementioned conditions [41,42,43,44]. Without this, there is a greater risk of inappropriate guidelines or guidelines that do not secure the “buy-in” from the target population. The aim of this study, therefore, was to explore the barriers, facilitators, and solutions to PA/E, sport, and nutrition in PKU from different stakeholder perspectives.

## 2. Materials and Methods

### 2.1. Sampling and Recruitment

A purposive sampling approach was employed, whereby all participants either had a formal diagnosis of PKU and were over 18 years of age, or caring for someone with PKU (<18 years of age), or were employed in a role related to the treatment of PKU (i.e., clinician or industry professional). These stakeholders were recruited through in-person conferences, social media, and snowball sampling (i.e., word of mouth). As a result, 75 individuals (*n* = 31 individuals with PKU, *n* = 13 caregivers, *n* = 17 clinicians, and *n* = 14 medical industry professionals) participated in this focus group study (Table 1). All participants provided written informed consent, and the study received institutional ethical approval.

### 2.2. Reflexivity and Positionality

The research team itself consisted of academics and professionals in both Sport and Exercise Science as well as Inherited Metabolic Disorders (IMDs). As a research team, being aware of our behaviour, positionality, values, and assumptions helped understand how this may affect data collection, analysis, and interpretation. Additionally, taking a qualitative neo-positivist approach [45] meant we were seeking to represent the views of the participants whilst minimising the impact of the researchers’ subjectivity. All researchers in the team have experience in research with elite athletes and/or with individuals with PKU. This allowed for shared learnings and approaches to suit the target population to be considered in regular group discussions. The lead author (AGS) surpasses the UK Chief Medical Officers’ Physical Activity Guidelines [33], whilst also having PKU, which might have affected how participants related to AGS, as a researcher and how much participants were willing to share. It may have made participants with PKU reveal more information or engage more in the research, as they have potentially built a stronger rapport between AGS, as a researcher, and them, as a participant, and possibly felt more understood compared to a non-PKU researcher [46]. Research has shown that the establishment of rapport is needed to generate rich data and personal information, whilst keeping respect for the participant [47]. As a researcher, the position of AGS cannot be changed; they recognise their role as an insider and how this may help to generate more in depth information. AGS is unable to be impartial as a researcher but instead was seen as part of the group.

### 2.3. Data Collection

A total of 13 (7 in-person; 6 online) semi-structured focus groups (FGs) were conducted with the range of participants consisting of 2–9 people per focus group [48]. This is in line with recommendations for focus group size to maintain rich, in-depth data and balanced group dynamics [49]. FGs were used for this study as they are considered a respected qualitative research tool to gain insight and valuable information within a field of interest [50]. The FGs covered three main areas, focusing on barriers, facilitators, and solutions to PA/E. The FGs were either held in-person at a national conference (The National Society for Phenylketonuria (NSPKU), Warwickshire, UK, 2024) or academic institution (Birmingham City University) or held online (via Microsoft Teams). The use of online FGs was seen as advantageous to gain data from those who could not attend the conference, were based overseas, or felt any environmental barriers preventing their involvement [51]. Prior to implementation, the FG questions and conduct of the FG was piloted with members of the research team. The FGs were recorded via MS Teams and transcribed automatically. The lead researcher (AGS) checked the transcription against the recording where appropriate. Each FG lasted approximately 45–60 min with 15 min allocated to cover each of the 3 questions and 10–15 min to discuss ground rules. Following the FG, the research team met and reflected on each session, discussed any key themes raised from responses, and adjusted the approach if necessary for future FGs.

### 2.4. Data Analysis

Following the FGs, the lead researcher (AGS) read through all the transcriptions and used thematic analysis [52] to map the responses onto the COM-B model of behaviour change [53]. The COM-B model is a recognised behaviour change model that proposes that behaviour (B) is impacted by the individual’s perceived capability (C) (physical and psychological) to take part in the behaviour, their motivation (M) (automatic and reflective) to undertake the behaviour, and their perception of opportunities (O) (physical and social) to take part. Initially, the lead researcher carried out coding of the data. A mind map was developed by the lead researcher (AGS) and shared with a member of the research team (LAG) along with the transcripts of the focus group. Once reviewed and agreed, two researchers (AGS and KL) met, and the codes were then grouped according to the COM-B model; where similarities were present, they were put together to form the overarching themes and sub-themes. This data was discussed within the whole research team to refine the specific themes, provide clear definitions, and ensure no data/themes were missed. This process was used to help take the raw data to actionable, theory-informed insights mapped to behavioural determinants [54] and has been used in previous research [55]. Finally, in mapping to the COM-B model of behaviour change, this may influence future intervention research.

## 3. Results

The total composition of focus groups and participant information can be seen in Table 1. Interpretation of the data analysis generated five main themes within the barriers and facilitators in relation to individuals with PKU performing PA/E, sport, and nutrition. These were as follows: 1. physical effects of PKU; 2. psychological effects of PKU; 3. support; 4. knowledge and management; and 5. opportunities. These will each be discussed in relation to the COM-B model [53] (Table 2).

### 3.1. Barriers and Facilitators

The following section outlines the focus group findings from each theme and sub-themes identified showing how each stakeholder perceived them to be barriers or facilitators to individuals with PKU engaging in PA/E, sport, and nutrition. These themes are also linked to the COM-B model throughout [53].

***1.***  ***PHYSICAL EFFECTS OF PKU (Capability)***

Within this theme, two further subthemes were identified: 1. metabolic control leading to inadequate energy perceived to be due to Phe levels and 2. long-term health concerns which were driving a need to be fit. Timing food and protein substitute intake accordingly around activities and ensuring individuals with PKU are eating enough to support PA/E was another commonly reported barrier by all stakeholders. A few stakeholders, including individuals with PKU and in the industry, related this in response to gastrointestinal issues (GI) occurring with ingestion of protein substitutes.

The second sub-theme was individuals’ concerns over their long-term health which facilitated their engagement in PA/E and sport. This was expressed from all perspectives, with adults specifically focusing on maintaining bone health, body composition, and weight-management strategies. Importantly, maintaining stable Phe levels was reported to help facilitate PA/E, sport, and dietary adherence and was a motivator to being healthy. Individuals with co-morbidities, such as osteoporosis alongside PKU, found PA/E helped manage these conditions, and industry participants also echoed this from their experience of working with individuals with PKU.

***2.***  ***PSYCHOLOGICAL EFFECTS OF PKU (Motivation)***

Preventing poor or maintaining good mental health is a large part of PKU management from living with a chronic disease and was a key finding from this study. Further sub-themes included the cathartic effects of engaging in PA/E and sport; both improved and reduced self-confidence around engaging in PA/E and sport with PKU and increased and decreased anxiety around engaging in PA/E and sport with PKU. For some individuals, there was a mental barrier for engagement either by having a chronic condition or receiving negative comments from others around them taking part. As a result, this reduced their motivation for PA/E and sport, stemming from difficulties with anxiety and self-confidence to initiate participation. Additionally, fear of judgement/comments from others and reduced belief about individual’s own self-efficacy impacted negatively on PA/E participation and sport performance. Individuals with PKU and clinicians shared their experiences of self-confidence and difficulties when trying to engage in PA/E, due to mental barriers and lack of self-confidence, which fed into their sport performance. This also included the fear individuals had themselves of initiating exercise, as they felt concerned of the unknown effects this may have on their metabolic control and consequently their PKU management. Individuals with PKU raised this as a barrier to PA/E and sport participation, and the lack of information and research around exercise and Phe levels reinforced this issue. Consequently, this acted as a barrier to even begin PA/E and sport from a perceived inability due to having PKU.

Psychological facilitators were also evident as a key facilitator for participation in PA/E. Individuals were motivated to engage in PA/E and sport for the cathartic effects felt afterwards, citing improved self-confidence and reduced anxiety, which helped their overall mental health and well-being. This was also expressed by both clinicians and industry professionals. The cathartic effects of activity included feeling happier post-exercise, feeling more in control, and improvements in overall well-being, which were expressed by all stakeholders as important to individuals with PKU. PKU adults, caregivers, and clinicians working in PKU reported the importance of participation for fun and enjoyment, and caregivers also reinforced this concept. The ability to escape from everyday life, stresses, or thinking about PKU was felt as a facilitator for PA/E and sport by many adults with PKU but also recognised by industry. A striking number of respondents expressed their mental health to be at the forefront for engaging in PA/E and sport. The improvements towards feelings of anxiety, self-confidence, and overall functioning facilitated by engagement in PA/E were also expressed by all stakeholders as being important, with patients specifically reporting it to be part of their own identity.

***3.***  ***SUPPORT (Opportunity and Motivation)***

The main theme of support covered a range of different sources, as shown in the sub-themes identified: 1. role models within PKU, 2. clinical, 3. family, and 4. peers. A lack of PKU role models participating in PA/E and sharing their experiences was identified as a barrier to engagement, which can be linked back to self-confidence and belief in one’s own ability. This was supported by all stakeholders, who discussed a lack of inspiration from those within the community, thus reinforcing the barrier towards engaging in PA/E and sport with PKU. Industry also expressed how the PKU population role modelling PA/E and sporting behaviour would increase motivation and beliefs around participation, which was agreed by all and seen as a fundamental but was an uncommon facilitator in the PKU community. A concept raised by clinicians and expressed by adults with PKU was the ability to relate to those who are already sharing their exercise journey with PKU through social media. Clinicians shared how individuals who may not want to or do not have the necessary experience to complete a particular event, distance, or time possessed a negative attitude, which acted as a barrier towards engaging in PA/E and sport. Furthermore, industry and clinicians stated barriers shown by parents where they are either *“*overprotective” of their child with PKU and think of exercise as an additional challenge so they do not engage or they “overcompensate” and are very encouraging and dedicated to exercise training regimes. It is clear from these findings that the barriers and facilitators for individuals with PKU will differ depending on individual circumstances, which highlights the need for more personalised approaches to PA/E, sport, and nutrition.

Support was shown to be a great facilitator for both adults and children with PKU in participating in exercise, from the friendships they created. All stakeholders supported this concept, as friendships appeared to make exercise encouraging and contributed as a large motivator for engagement. Peer support from close friends who understood PKU, family members, or partners who were also active was a key concept supported by all stakeholders to facilitate regular participation by individuals with PKU. Adults expressed how parental engagement in PA/E motivated them to be active with PKU. The main reoccurring message shared by all was the importance of individuals wanting to “fit-in” and feel “normal”, linking back to self-confidence. Interestingly, industry states that the general greater awareness of PA/E in the non-PKU population has facilitated a great interest in individuals engaging in PA/E, sport, and nutrition.

***4.***  ***KNOWLEDGE AND MANAGEMENT (Capability and Opportunity)***

The overarching theme that resonated with all stakeholders was a lack of knowledge regarding PA/E, sport, and nutrition. This fundamental knowledge gap reported by all can be linked back to the issues with the support systems available to the PKU community, which links to the identified subtheme 1. lack of awareness in the PKU community. The impact on PKU care was clear, with several adults expressing how they have never discussed PA/E or nutrition with PKU in clinic appointments. Some individuals shared they have even changed things without their clinic’s consent to align with their goals, confirming the inconsistencies within the support systems and misalignment amongst stakeholders. Responses from industry also expressed how they were unaware of any reason why someone with PKU would struggle to engage in PA/E or sport (please see quote in Table 2) and expressed more interest in the area in recent years. However, clinicians (and individuals with PKU) acknowledged a lack of conversations regarding PA/E, sport, and nutrition. This displays a disparity between stakeholders and their experiences with patients, showing that individuals with PKU seem to be seeking outside of clinic support for PA/E and sport. This limited knowledge was also evident in relation to fuelling sporting performance, with individuals reporting that they were uncertain if their nutrition was correct for the demands of their exercise and whether this would impact their Phe levels, describing it as a “guessing game”. This suggests an unmet need for nutritional support to maintain metabolic control and a lack of clarity on how to optimise performance outcomes in PKU. This is not surprising given the lack of literature investigating the Phe response to exercise [56,57].

Increased education to improve general knowledge and management strategies of PKU was perceived as a facilitator to participation, which was communicated by all stakeholders. Adults expressed how having more information and conversations around this topic made PA/E and sport appear more desirable. Clinicians and industry both agreed that if you were from a more privileged background and were well educated (i.e., degree level), this aided the understanding of the importance of PA/E, sport, and subsequent participation. In comparison, underprivileged and less well-educated individuals or families were less likely to prioritise PA/E, sport, and nutrition. This is supported by research where higher Phe levels have been found in those families who are less educated and therefore are less likely to understand the importance of PKU management with PA/E and sport participation [58]. The need for specific advice for individuals with PKU was apparent and of high significance to all stakeholders, with clinicians suggesting that if research can be conducted to show a positive effect of PA/E on metabolic control, it may help motivate patients to participate. This was also raised in relation to the patient’s own self-belief regarding involvement in PA/E and sport, alongside their metabolic control and attitude towards PKU. Clinicians reported that those who are well controlled and have a positive attitude towards their PKU are usually the ones regularly taking part in PA/E and sport, whereas individuals expressed that also having the ability to have open conversations with their metabolic team regarding any dietary changes to support exercise goals was a facilitator and provided a source of support and motivation. Many individuals reported poor experiences of trying to engage with clinics, sharing that the topic of PA/E, sport, and nutrition to support this is often dismissed in conversations, linked to cost and/or a lack of knowledge. Overall, the knowledge and management of PKU was a key area in relation to the barriers and facilitators to PA/E and sport participation, with a high number of responses from all stakeholders around this theme.

***5.***  ***OPPORTUNITIES (Opportunity)***

Sub-themes reported were 1. availability of sports to engage in, 2. cost, and 3. influence of socioeconomic demographic status on the availability of sports and costs. This lack of opportunity to engage with sports was reported by clinicians working in PKU, with social deprivation and financial costs as barriers to engagement. Sociodemographic and economic status were influential to being either a facilitator or barrier to participation in PA/E. This was supported by another clinician who reported that these opportunities were only available to those who could afford it. Those with higher incomes had the opportunity to try different sports clubs, and parents were found to be a key influence on childhood and lifelong participation in PA/E. In addition, the logistical challenges of the Phe-restricted diet regarding opportunities to engage in PA/E was apparent in both patients and caregivers of those with PKU. Individuals with PKU and caregivers described how the timing of exercise (both scheduled classes or preferred time of exercise) with protein substitute intakes or mealtimes across the day, specifically early mornings or after work/school, provided difficulties. This presents the challenge of having the opportunity to engage in sports but at an appropriate timepoint so that dietary management is still aligned. Here, the dietary management of PKU, together with the time available for participation in PA/E and sport, was reported as a barrier to participation.

### 3.2. Solutions

Data analysis using the highlighted five main themes from different stakeholder perspectives identified various solutions for individuals with PKU to engage in regular PA/E, sport, and nutrition. These will each be described in turn below in relation to the COM-B model [53]. Please see Table 3 for each of the five key themes, subsequent sub-themes, and relevant transcript quotes, which have been aligned with the COM-B model of behaviour change [53].

***1.***  ***PHYSICAL EFFECTS OF PKU (Capability)***

A solution to participation in PA/E and sport was metabolic control and being able to use PA/E and sport to stabilise Phe levels, with the reassurance that involvement would not be detrimental to overall control. Clinicians working in PKU reported this to be of high significance for patients and a facilitator to engaging in activity. Good metabolic control was indicated to help overall performance in daily functioning alongside sport performance, if the latter was of interest. A fundamental part of achieving this is adherence to the Phe-restricted diet; however, individuals with PKU described experiencing GI discomfort and feeling sick from special low-protein prescription food and/or protein substitutes. As a result, improvements in palatability and tolerability of special low-protein prescription foods and protein substitutes were suggested. Individuals with PKU expressed that once this has been resolved, it may aid more regular involvement in PA/E and sport.

***2.***  ***PSYCHOLOGICAL EFFECTS OF PKU (Motivation)***

A solution to the psychological barriers was having the correct, tailored support and evidence so they could confidently participate in PA/E and sport, which was cited as lacking from all stakeholder groups. Adults with PKU stated how more detailed and specific information, especially regarding metabolic control, could help those with PKU feel more confident in participating in PA/E and sport. As a result, this could increase the chance of reaching out to someone, e.g., a personal trainer (PT), clinician, or sport coach, with an understanding of PKU, PA/E, and sport. Individuals also expressed how they want to be able to thrive and peak with PKU and not just manage the condition to reduce any harm. Clinicians thought that PKU management would influence overall motivation and that having control over Phe levels would act as a solution to engagement in PA/E and sport. This holistic approach was voiced to be important to all stakeholders. A consideration for stable Phe levels and tailored information was the driving force for increasing motivation and confidence whilst reducing anxiety around participating in PA/E and sport. Individuals shared a lack of confidence around exercise, which also links to the limited overall knowledge, awareness, and support in PKU clinics. Increasing accessibility to participation was seen as a key solution to reduce anxiety in patients from a clinician’s perspective. Provision of PKU-specific advice and awareness of this in the community, the potential for exercise prescription, and a safe place to exercise to help reduce anxiety around involvement in PA/E were all seen as important solutions by all stakeholders.

***3.***  ***SUPPORT (Opportunity and Motivation)***

A major feature described by all stakeholders was the influence of support in PKU management. The main message was a sense of community across all ages for support and guidance with PKU management. Another key solution suggested by all stakeholders was the visibility of role models who have PKU but also participate in PA/E and sport, which they suggested use of social media may help with. Individuals reported how useful it was to see others with PKU undertaking PA/E and sport and that it provided individuals with a sense of “motivation” with people “sharing their experience starting from scratch”. Industry and clinicians also supported using social media to improve individuals’ and caregivers’ overall confidence around PA/E and sport with PKU and more people sharing this to help promote within the community.

Industry recognised how education plays a fundamental role in PKU support and how this should be encouraged from an early age. Receiving support from peers, coaches, PTs, employers, and GPs was also deemed necessary for patients and caregivers, with resources to help educate others on PKU around PA/E, sport, and nutrition. Clinicians echoed this need for simple fact sheets to aid clinical conversations like those presented in other chronic conditions, e.g., diabetes [59]. The knowledge and management of PKU linked closely with support, and this went hand in hand with overall participation in PA/E and sport.

***4.***  ***KNOWLEDGE AND MANAGEMENT (Capability and Opportunity)***

The fundamental factor underpinning both the barriers and facilitators to engagement in PA/E, sport, and nutrition overall was the knowledge and management of PKU. This was echoed from all stakeholders from the vast quantity of responses given regarding the solutions to participation (please see Table 3). Individuals with PKU expressed how specific guidelines, advice on the best modes of activity for metabolic control, and nutritional support, including tailored recommendations, e.g., timing of protein substitutes, would be helpful solutions. Clinicians proposed the idea of a potential sport protein substitute, and industry also raised this, suggesting that new products could be made to support PA/E and sport. Clinicians also expressed how provision of a resource centre providing PKU-specific information to which they could signpost patients would be another welcomed solution. Resources for employers, PTs, GPs, clinics, caregivers, and individuals with PKU to educate those on the knowledge and management of PKU with PA/E, sport, and nutrition was considered an essential solution stated by all and was closely linked to the support systems mentioned above. Individuals reported inconsistencies in PKU care, as mentioned in the above *barriers* section, and how important education for the clinical metabolic teams was.

Individuals with PKU voiced the importance of having open 1-2-1 consultations/discussion in clinical appointments and being mindful to dietary changes to support activity, allowing this topic to be normalized within the community. Individuals wanted to excel with PKU, not just survive. Clinicians admitted that the lack of discussions with patients presented an unmet need in the community, but current clinical barriers of restricted time and information need to be addressed. Industry agreed that there are missed opportunities within clinic appointments for group sessions around education and engagement in PA/E with PKU. Additionally, all stakeholders including clinicians voiced how education within the metabolic team would help provide this tailored information and ultimately support patients. Advancements in knowledge and a step-by-step guide were also deemed essential to delivering this. For example, research into exercise with diabetes has led to multiple resources being developed, including the development of the “Type 1 and Type 2 Diabetes Exercise Action Plan” [59]. This advises individuals with diabetes on how to manage their condition, with specific guidelines for commencing exercise and recommendations for both during and after exercise included.

***5.***  ***OPPORTUNITIES (Opportunity)***

Being able to access more sporting opportunities within local communities, PKU events, clubs, or activity schools was agreed by all stakeholders to be of key value for engagement in PA/E and sport across all ages. Clinicians reported how deprivation and poor financial status are linked to limited opportunities and how more weekends away for those with PKU would help provide the “opportunity” for engagement in sporting activities. Individuals and caregivers reported how the recent inclusion of PA/E, sport, and nutrition in PKU-specific conferences (e.g., NSPKU) and events focused on this, and these have and would help provide an “opportunity” for engagement and increased knowledge on how to manage PKU with PA/E and sport. Individuals also suggested specific PKU educational PA/E-, sport-, and nutrition-based retreats/camps, and industry also raised how national PKU conferences should have a focus on PA/E. All stakeholders reported that workshops (both online and in-person) focusing on this topic would help PA/E, sport, and nutrition with PKU, plus tailored resources to facilitate some of this discussion. Consequently, something comparable yet specific to PKU requirements may aid safe participation in PA/E and sport in individuals with PKU. Whilst these proposed solutions have credit, it should be a priority to provide low-cost opportunities for patients to reduce the barrier of cost to engage in PA/E and sport.

## 4. Discussion

This is the first study to explore the barriers, facilitators, and solutions to participation in PA/E, sport, and nutrition across a range of stakeholder perspectives, including individuals and caregivers of those with PKU, clinicians, and industry professionals working in PKU. Five main themes were identified: 1. physical effects of PKU; 2. psychological effects of PKU; 3. support; 4. knowledge and management; and 5. opportunities. The primary finding revealed that misalignment was evident between individuals with PKU, caregivers, clinicians, and industry professionals. Several clinicians and industry representatives did not think PA/E or sport would be physically more challenging for those with PKU compared with the general population. In contrast, individuals with PKU and their caregivers stated fatigue, poor recovery, low energy, and fear around the impact of exercise on blood Phe control were barriers to PA/E and sport. Individuals with PKU expressed how participating in PA/E impacted positively on their mental well-being, daily functioning, and happiness, improving their self-confidence, PKU management, and long-term health. The social aspect of PA/E and sport was key to participation together with knowledge and information to support PKU management. Identified solutions to PA/E and sport participation were generally education-related, including greater knowledge regarding the impact of PA/E on metabolic control, improvements in the composition of special low-protein foods, tailored specific PKU nutritional advice for optimisation of sport performance, opportunities to safely participate in PA/E and sport, more awareness of PA/E and sport within and outside the PKU community, specific PKU guidelines for PA/E, sport, and nutrition, scientific research, and PA/E- and sport-related community events.

***1.***  ***PHYSICAL EFFECTS OF PKU (Capability)***

Many of the barriers described by PKU were fatigue, mental health issues, co-morbidities, social isolation, and poor quality of life, which have been self-reported by patients in previous research [27,28,29]. Additionally, the physiology and psychology of metabolic control was an apparent barrier. Having high Phe levels caused individuals with PKU to perceive more fatigue and tiredness and be less likely to be motivated or energised to engage in PA/E or sport. High Phe levels have been shown to have a direct negative effect on both mood and neuropsychological performance (specifically sustained attention) in adults with PKU [60]. Furthermore, fatigue was reported by patients which was heightened post-exercise, with energy crashes being regularly experienced by those after PA/E reducing their “capability” for exercise. This fatigue response has also been found in previous research into individuals with PKU [19,20] and supported this study’s findings. Despite the well-known benefits of PA/E and sport on physical and psychological health in the general population [61], there is a lack of understanding of how PA/E, sport, and nutrition impacts individuals and athletes with PKU. In this study, the energy drops reported by individuals with PKU post-exercise could be attributed to potential nutritional deficiencies, specifically in certain vitamins and minerals, which can be apparent because of compliance and access issues with protein substitutes and the Phe-restricted diet [62,63,64]. The composition and access to protein substitutes and special low-protein prescription foods was a reported barrier for participation in PA/E and sport and could therefore explain the compliance issues. Individuals with PKU and caregivers expressed how increased hunger, reduced satiety, and the logistical challenges of timing PA/E and sport around protein substitutes and mealtimes to prevent any GI issues was a frequent battle. Improving palatability and tolerability of protein substitutes and special low-protein prescription foods could therefore aid participation in PA/E and sport. However, poor compliance to protein substitutes could reduce the intake of essential vitamins and minerals which are key components of metabolic enzymes. This could potentially disturb the activity of vital enzymes of intermediary metabolism, which in turn could negatively affect the energy levels of individuals with PKU with poor compliance [65]. Furthermore, another reason for this fatigue reported may be due to an increased oxidative stress and inflammation response which is often seen post-exercise [66]. High Phe levels and poor compliance with the Phe-restricted diet have been associated with reduced antioxidant defences in individuals with PKU [17], which may suggest poor diet compliance may be a barrier to PA/E and sport [65]. Further research is needed to confirm whether the fatigue commonly reported, especially post-exercise, is a consequence of energy dysfunction in PKU, the composition and compliance with the Phe-restricted diet, increased oxidative stress and inflammation, or something else. Consequently, at present, this is acting as a barrier to engagement in PA/E and sport for individuals with PKU.

Patients expressed their concerns regarding the physical effects of having PKU and their long-term health, specifically bone health, body composition, and weight management, which were a driving force for participation in regular PA/E and sport. Research has shown that individuals with PKU are potentially at risk of poor bone health [23,33], poor muscle mass [67], poor BMI status [68], and overweight/obesity [30,69,70]. Individuals with PKU reported using PA/E and sport to try to offset any potential co-morbidities to sustain their health as they age. Equally, for those individuals who did experience additional co-morbidities or health issues, they found PA/E and sport helped manage their symptoms. As a result, future interventions should look to incorporate regular PA/E and sport into an individual’s weekly routines to help complement their PKU management and aid their long-term health. However, without the specific research, particularly on bone health, this remains a challenge and a “guessing game” for the PKU community.

Furthermore, individuals shared how having stable Phe levels and compliance to the Phe-restricted diet facilitated participation in PA/E and sport and helped them feel healthy overall. The added metabolic challenge seemed to “motivate” certain individuals to overcome this barrier, and it emphasised the importance of building the clinical team relationships with patients/caregivers to manage PKU and any symptoms, e.g., fatigue alongside PA/E and sport. Research has shown a significant effect of the patient–clinician relationship, specifically communication skills, improving results of care in adult patients [71]. Ideally, patients and clinicians could co-design a tailored nutritional plan to support PA/E, sport, and nutrition to optimise their Phe levels whilst carefully managing the fatigue response, energy levels, and recovery. Yet, for this to be implemented successfully, first, we need to consider the current challenges raised from this study. This included the present lack of clinician knowledge and available resources focused on PA/E, sport, and recovery with PKU. This may explain why individuals with PKU generally feel unsupported to engage in PA/E, sport, and optimal nutritional strategies. Based on this evidence, research and guidance should work on giving individuals with PKU the physical and psychological “capability” to engage in PA/E and sport [53].

***2.***  ***PSYCHOLOGICAL EFFECTS OF PKU (Motivation)***

Mental well-being was considered a facilitator and source of “motivation” for individuals with PKU in engaging in PA/E and sport. The positive effects of exercise in feeling better were perceived a major factor for improving daily well-being and motivation for PA/E and sport. Living with PKU has shown to affect the mental health of individuals with PKU, with 52% of UK adults with PKU (*n =* 332) reporting difficulties with depression or anxiety [27]. This is much higher compared to the general population, such that an international systematic review including over 140,000 individuals reported the prevalence of anxiety to be 27%. Based on the findings of [27] and the current study findings, it can be argued that adults with PKU have a greater number of perceived barriers than non-PKU cohorts. These findings are despite many adults with PKU reporting how they are “motivated” to use regular PA/E and sport as an escapism from everyday life, which included PKU management, regardless of their overall metabolic control. Adults with PKU also reported how they felt happier in themselves, less anxious, and more confident after PA/E or sport participation. Consequently, regular PA/E or sport may help to manage both physical and psychological health, acting as a beneficial tool for PKU management and a “motivating” factor for participation. Yet, the need for psychological support needs to be readily available for all regardless of clinic location, as stated in the recent EU guidelines [15]. This could help individuals have more control over their mental health and well-being, as most UK PKU clinics have little psychological input. Research has shown many adults with PKU feel their symptoms or issues are not always taken seriously by clinicians, with only one in four patients (*n* = 631) receiving psychological support [27]. However, all stakeholders reported that more psychological support for individuals with PKU was a much-needed solution, alongside overall education on PA/E, sport, and nutrition to reduce anxiety and aid their confidence to engage in PA/E or sport.

***3.***  ***SUPPORT (Opportunity and Motivation)***

A key part of PKU management was the perception of quality care and support that both individuals and caregivers of those with PKU received. Support came from a variety of sources including peers, family members, caregivers, sport coaches, PKU clinicians, or other individuals with PKU. Industry provided a broad approach but were a limited source of support in regards to PA/E, sport, and nutrition with PKU. Both clinician and wider population education on the knowledge and management of PKU regarding PA/E and sport was stated as fundamental to feeling supported, potentially because it could help alleviate fears of participating in PA/E and sport and maintaining metabolic control. The ability for individuals with PKU/caregivers to have discussions regarding PA/E, sport, nutrition, and PKU management with various people, e.g., PTs, peers, and clinicians, and have tailored information available was strongly linked to the support they felt. Additionally, the sense of community was apparent to PKU management and not feeling alone. Solutions were to have PKU role models engaging in PA/E or sport share their experiences and journey for others to see both in-person and online (e.g., on social media). This was perceived as a source of “motivation” and inspiration to others with PKU to engage in various forms of PA/E or sport, but also as an educational aid to show how they could manage their PKU to support the demands of activity. Social media has been found to be effective at making positive changes to physical activity and diet-related behaviours in the non-PKU young adult population and subsequent participation in PA/E [72]. However, a lack of PKU role models could be perceived to reduce this impact and the overall support felt within the PKU community.

Family and peer influence for participation in PA/E, sport, and nutrition was clear and important for “motivation” in both adults and children. Previous research also echoes this, as children with parents who are regularly physically active, eat healthily, and are a healthy weight were more likely to have children who replicated this behaviour [73]. Social deprivation is a large factor which may prevent this within families (both within and outside PKU households), so regular PA/E or sport may be more difficult due to low income and time pressures. Consequently, even if PKU caregivers and families want to engage in healthy behaviours, they may have limited resources available to do so, thus reducing the “opportunity” for PA/E or sport. This is where clinics and local communities could help to educate families with low-cost approaches, e.g., home workouts or local free sports classes/events providing “opportunity” for PA/E and sport [16]. It is worth noting, however, that these approaches may be less impactful in areas of large deprivation, as facilities are usually of low quality and opportunities for social PA/E or sport are limited. Additionally, clinicians and dieticians within the PKU community could act as role models for healthy behaviours. Research in other areas has shown physically active physicians and health care providers, including dieticians, were more likely to provide advice on PA/E to their patients and be seen as powerful role models for PA/E [74]. Individuals with PKU did not express whether they knew the activity or diet behaviour of their clinicians; however, if they believed them to be active, this could potentially align as another support system via role modelling and providing “motivation”.

***4.***  ***KNOWLEDGE AND MANAGEMENT (Capability and Opportunity)***

The overarching theme of knowledge and management underpinned and linked all the themes together. It was clear that a large gap in the knowledge and management of PKU in relation to PA/E, sport, and nutrition was the driving force for the inconsistencies in the support felt, the physical and psychological effects of PA/E, sport, and ultimately the “capability” and “opportunity” for participation. Having more research specifically on individuals with PKU would be key to unlocking this type of information. There is currently very minimal experimental research on PA/E or sport in individuals with PKU [57], which is likely due to limited funding and reluctant uptake from participants. All current guidelines also are focussed on general population data for PA/E, sport, and nutrition [34,35,37], which may deter individuals from undertaking the exercise due to fear of exacerbated fatigue and energy crashes. In the current study, individuals with PKU and caregivers reported not feeling confident in managing their PKU around PA/E and expressed concerns regarding their metabolic control, which is essential for their long-term health. This is surprising given a nationwide UK-based survey that also showed 86% of individuals with PKU on a Phe-restricted diet were concerned about their long-term health [75]. Based on this evidence, there is a clear need to determine the appropriate PA/E, sport, and nutrition advice for individuals with PKU, which will likely come from scientific research. Once this is available, educational resources around PA/E, sport, and nutrition could then be applied in practice, which may be delivered by clinicians, charities, and/or industry professionals, providing the “capability” for PA/E. Previous research has shown the success of using co-production in such interventions in non-PKU populations for increasing PA/E [76,77].

Furthermore, increasing knowledge to provide the “capability” for PA/E, sport, and nutrition may be one way to help encourage and maintain participation in individuals with PKU. Regular consultations providing 1-2-1 advice were considered vital for education and practical application of knowledge. This would not only help with PKU management, sporting performance, and recovery but also help to develop a strong, supportive relationship with the patient’s clinical team, which has shown to aid health outcomes [71]. Further work is needed to discover how this could be implemented, however, as at least from a UK context, clinical PKU appointments are often short with large gaps of time between them, i.e., annually or longer [78]. There is also the question of where this advice should come from, as healthcare services have large demands and reduced scope for increased roles. However, the adoption of exercise sciences is increasing in UK healthcare, with specialists in other areas such as obesity and various rehabilitation areas (e.g., stroke and long COVID) [79]. Outside of healthcare, this may be an area that charities and/or industry professionals who are working in PKU may consider introducing.

The PKU community has recognised the need for more information and support around PA/E, sport, and nutrition and expressed how consistent approaches both from clinical teams across the world and within different areas are necessary. Although there have been many advancements in protein substitutes [80], a sport-based protein substitute has not been developed, particularly to help post-exercise fatigue symptoms reported. Additionally, the potential for a centralised hub of information specific to PKU management with PA/E, sport, and nutrition adjustments was a popular solution. These innovations could provide the “capability” and “opportunity” by supporting both individuals and their families to engage in PA/E through events dedicated to PA/E, sports, and nutrition with PKU. Consequently, the provision of PA/E opportunities through clinical teams and national and international PKU organisations would only advance the level of PKU care.

***5.***  ***OPPORTUNITIES (Opportunity)***

Solutions for improved PKU management in relation to PA/E, sport, and nutrition points towards these factors being more accessible to patients, but also for this to be seen as a critical part of their clinical care. This can be achieved through education and conversations on lifestyle factors, such as PA/E, to patients from outside and within clinical care. This may subsequently alleviate fears of PA/E or sport and as such provide the opportunity to take part. For example, as briefly mentioned above, education programmes have been developed especially for clinicians to allow encouragement and safe recommendations for PA/E to their patients with T2D [81]. Similar resources have been developed for patients with multiple sclerosis (MS) [82], asthma [83], and chronic obstructive pulmonary disorder (COPD) [84]. Low-cost options to increase participation would include developing online webinars and physical resources, in addition to clinicians or fellow members of the PKU community sharing locally led PA/E activities. Ultimately, the aim would be to provide more opportunities for regular PA/E and sport participation regardless of individuals’ or families’ sociodemographic or economic status. Having said this, providing in-person PKU events and workshops on the topic of PA/E, sport, and nutrition with PKU, with scheduled time for sporting endeavours, was a welcomed solution by all stakeholders. Suggestions from participants included weekly/monthly meet-ups to complete PA/E. Greater levels of knowledge and more focus on PA/E, sport, and nutrition within national and international conferences was also proposed, e.g., group exercise sessions and PA/E workshops, but this is constrained by financial costs and availability within conferences. Conversely, only those with higher incomes or positive parental influence/education would likely access these types of events, and individuals from lower-income backgrounds would potentially face financial barriers. A more inclusive solution would be to provide a combination of the above approaches to allow more opportunities for individuals with PKU to regularly participate in PA/E or sport.

### Limitations

In the current study, the higher number of females (*n* = 65) to males (*n* = 15) makes the data set more aligned to the female PKU population, despite the condition being autosomal recessive (i.e., not linked to sex chromosomes). This is likely due to the general pattern that females tend to participate in qualitative methods research compared to males who tend to gravitate towards quantitative studies [85], as well as the need for maternal support in females with PKU [15]. Using a combination of online and in-person focus groups allowed for greater overseas participation, but also provision of in-person discussions meant more data was likely shared, i.e., longer comments than typical of online focus groups [86]. A further limitation of this study is that little information on the individuals themselves with PKU in terms of their current blood Phe levels, physical and mental health, or their socioeconomic status was collected. This would have allowed us to provide more conclusive barriers, facilitators, and solutions for more specific groups of individuals with PKU.

## 5. Conclusions

This research highlighted that individuals with PKU and caregivers of those with PKU experience several barriers to PA/E, sport, and nutrition. Misalignment was evident between the reported barriers of individuals with PKU and the perspectives of clinicians and industry. A key finding of this study is that there are additional perceived barriers to PA/E, sport, and nutrition in individuals with PKU which do not seem to be echoed by the other stakeholder groups or what would typically be seen in the general population. Consequently, emphasis on increasing knowledge and understanding on the barriers and facilitators for PA/E of those with PKU could help collaborate stakeholders to provide more “opportunity” for participation. This study offers some solutions as to how the “offer” to individuals with PKU of PA/E, sport, and nutrition from industry, charities, and healthcare could be improved, although they require to be implemented and tested for suitability in future research. Based on the present study solutions, increases in knowledge and understanding are required, followed by further research to explore the responses to PA/E. This should be followed with research on appropriate nutritional strategies to support PA/E and sport in PKU.

## Figures and Tables

**Table 1:** The composition of focus groups.

13 Focus Groups (7 In-Person; 6 Online)	*n* = 75
Industry	14 Male: 2 Female 12
Individuals with PKU	31 Male: 7 Female: 24
Caregivers	13 Male: 4 Female: 9
Clinicians	17 Male: 2 Female: 15

**Table 2:** Identified main themes, sub-themes, and relevant verbatim quotes to display the barriers and facilitators of individuals with PKU performing physical activity, exercise, sport, and nutrition from key stakeholder perspectives.

Theme	Sub-Theme	Example Quote
**PHYSICAL EFFECTS** **OF PKU**	Metabolic control leading to inadequate energy due to Phe levels Long-term health concerns driving need to be fit *(Capability)*	***Barriers:*** *“Constant battle of just not having enough energy, not being able to fuel myself, not being able to recover properly, which definitely prevented me from reaching the level I wanted to reach in my sport.”* (PKU Individual) *“…some of the teenagers who maybe run very high levels, just extreme tiredness. That’s actually not with exercise, but they just get home from school or whatever, and they’re asleep and so just no capacity…absolutely nothing to do with exercise at all…run out of energy. Can just about cope going to school and that’s it”* (PKU Clinician) ***Facilitators:*** *“So, when she is good metabolic control, it definitely is a great facilitator.”* (PKU Caregiver) *“Additional health benefits… they may have other co-morbidities…but generally, to just improve your health.”* (Industry) *“…the younger ones might be more interested in like muscle building…ladies sort of in sort of menopause sort of ages and like they should be doing it for muscle strength, if for their mood and feeling better.”* (PKU Clinician)
**PSYCHOLOGICAL** **EFFECTS OF PKU**	Cathartic effects of engaging in physical activity, exercise, and sport Improved and reduced self-confidence around engaging in activity with PKU Increased and decreased anxiety around engaging in activity with PKU *(Motivation)*	***Barriers:*** *“…I think a lot of it is for me is it’s mental now, that I’ve always gone in the back of my head, I can’t do it, I’m not going to be able to do it because I’ve had so much people seeing negativity to me throughout my childhood. And I stopped dancing because people were then saying stuff…”* (PKU Individual) *“I think sometimes they don’t even believe exercise and similar things is an option to them anyway, because they’ve got a chronic condition. Therefore, they don’t believe they can do XY and Z, and exercise might be one of those things that they do even believe is an option to them that they can do it in a healthy, managed way.”* (Industry) *“A sort of twin anxiety, one is you’ve got to try something new and maybe meet other people that you don’t know and new people and interact with new people, but also you’ve got to navigate and go to a new place and a new environment.”* (PKU Clinician) ***Facilitators:*** *“…if they’re struggling with the diet and struggling with things that come with PKU, maybe it’s a bit of escapism as well.”* (Industry) *“…it just makes me feel happy…I just feel happier in myself once I’ve done that class…I find it enjoyable…and it’s just an hour in my life where I don’t need to think about work, I don’t need to think about the stresses of work, PKU, my life. I’m just in a bubble of enjoyment where I can just let go.”* (PKU Individual)
**SUPPORT**	Role models within PKU Clinical Family Peers *(Opportunity and Motivation)*	***Barriers:*** *“…it’s like important to almost like have a role model or see people do it because then you actually think, oh, I can do this too.”* (PKU Individual) *“social media is good for people being active because they see other people being active. But sometimes I think it has a negative effect…I can’t do a one hour 44 half-marathons, so I’m not going to bother to do it in five hours, so I think social media definitely has an influence in the younger adult population…”* (PKU Clinician) *“…there’s other peers who don’t have an understanding of PKU…. she’s like, well, maybe I’m not a sporty person…so she’s looking for other avenues to identify with and to understand who she is. And I’m really trying to support her around that, but I think that’s hugely difficult at times.”* (PKU Caregiver) *“Sometimes parents are overprotected with PKU children. In particularly say that where there’s been significant trauma around how the parents have received the diagnosis. And you know they see the child as everything being unfair…if exercise is layered on top as something else that they have to do, then that’s something they might push back on. So, it’s a parental kind of barrier…”* (Industry) ***Facilitators:*** *“I think also the friendships you make on the way, you do meet supportive people. I think I’m finding that the same and it kind of encourages you to say if you take part in the competition or you take part in a half marathon that’s something that’s super rewarding whether you have PKU or not that sometimes it can feel extra good knowing that I have PKU and I’m doing this.”* (PKU Individual) *“And we’re very physically active family, so I think that too is definitely a facilitator.”*(PKU Caregiver) *“…if there’s more exposure to information online, peer-to-peer seeing you know seeing other people talk about or doing sporting activity and maybe you know even better saying how they compensate or don’t need maybe don’t need to compensate for doing activity with PKU, then I think that’s going to be a motivating factor.”* (Industry)
**KNOWLEDGE AND** **MANAGEMENT**	Limited knowledge of PKU with physical activity, exercise, and sport Clinical awareness of PKU Public awareness of PKU Awareness in the PKU community (*Capability and Opportunity*)	***Barriers:*** *“I think their main barriers are probably the same as the general population…”* (Clinician) *“…I can’t think of a scenario where someone with PKU had specifically raised barrier in relation to PKU about doing some form of physical activity…”* (Industry) *“I have a feeling that a lot of research needs to be done…whenever I ask for example can I drink creatine ‘cause I do weightlifting a lot in past 4 years and I do it quite heavy. I can do way above my body weight, and I feel like I need some creatine or something…I’m training 4 to 5 days every single week 4 sessions of 2 h and I’m lifting weights every single week and you tell me that I don’t need it. I’m not a professional so to me that’s sort of a barrier of knowledge because I feel like my body wants something extra…”* (PKU Individual) *“When we’ve talked about her, perhaps not having enough protein supplement and does she need more, we’re always told no, it’s absolutely fine…I think psychologically, she feels like it’s going to make her not feel very good and then that kind of puts her off…joining in and putting the effort in to do the exercise.”* (PKU Caregiver) *“There are inconsistencies across the board.”* (PKU Individual) *“I think I probably don’t focus on that. I kind of focus on things like, yes, you’ve got lots going on, haven’t you? I wouldn’t really talk about physical exercise very much unless they’re really struggling mentally actually…I tend to bring in physical exercise mostly as a weight control conversation or a weight management conversation.”* (PKU Clinician) ***Facilitators:*** *“…recently I’ve had a chat with my dieticians and metabolic team…I did want to gain weight in the form of muscle, but my plans have kind of changed since then. But for the first time ever, they actually said why don’t we try giving you another supplement and we’ll see how it goes…And that kind of made me feel more like supported and motivated cause I’ve always kind of, I wouldn’t say dismissed the hospital, but I always felt a bit like they weren’t on my side, if that makes sense. But for the first time, as I had quite an open conversation with them…”* (PKU Individual) *“I would say education…movement doesn’t have to be going to the gym or running a marathon…I think unless you understand that, then it’s like, why should I bother? And so I think education is a really, really important aspect of you know, we’re facilitating someone too perform physical activity or just move in general.”* (Industry) *“I think maybe as well with PKU adults it depends how people feel about PKU as well. So we’ve got some adults who are kind of like I’m just going to do whatever I’m not going to let PKU you get in my way, but there’s other people who struggle with that.”* (PKU Clinician)
**OPPORTUNITIES**	Availability of sports to engage in Cost Influence of socioeconomic demographic status *(Opportunity)*	***Barriers:*** *“…we deal with is families who have and families who have not, and it’s really the have nots and some of it is some will say some live in areas where it’s not safe to go out in the evening and play or there’s no safe play areas for them to go and do exercise. I think the other thing is that the families who live in deprived financially and just everything else, they’re the ones who don’t get to the after-school clubs and the activity clubs, they just don’t get opportunity to do it…so I see a lot of lack of exercise and physical activity due to deprivation.”* (PKU Clinician) *“…trying to organise the times of swimming lessons or football lessons. Sometimes they’re not compatible with mealtimes or taking supplement time…”* (PKU Caregiver) *“…he wants to learn to doing karate…I might be struggling because I live right now far away…is 45 min walking.”* (PKU Caregiver) ***Facilitators:*** *“…And the lower the cost, so something that was cost free like going outside and just walking and talking or walking, walk, run, walk, run type thing.”* (PKU Clinician) *“…it is the parents and it’s kind of what, like social demographic they’re in. If they’re in like, you know like higher middle classes they’re the ones that all the kids are all doing all different sports, and they’re just to do things and they’re always at different clubs and things. So, they’re the ones that we see that are, you know, are motivated to get their kids doing lots of different things.”* (PKU Clinician)

**Table 3:** Identified main themes, subsequent sub-themes, and relevant example quotes to display the solutions to people with PKU performing physical activity, exercise, sport, and nutrition from key stakeholder perspectives including individuals with PKU, caregivers of those with PKU, clinicians, and industry working in PKU globally.

Theme	Sub-Theme	Example Quote
**PHYSICAL EFFECTS** **OF PKU**	Metabolic control leading to inadequate energy due to PHE levels Long-term health concerns driving need to be fit (*Capability)*	***Solutions:*** *“*…*but a lot of patients do have IBS…And I think there’s some evidence that walking and are kind of moving…and if you do a weight bearing exercise that can help there is there is some literature out there…”* (PKU Clinician) *“…if there were products that weren’t as harsh in your stomach or easier to travel…to be able to get energy if there is a way you could have something like, say, those energy bars that are quick to take, or you travel easily with them…”* (PKU Individual) *“…this particular person didn’t like bread, never ate bread, never ate low protein bread and didn’t like pasta we were stuffed really because we couldn’t get enough carbohydrate into her…But immediately she went out of control as far as the PKU was concerned and immediately a performance fell off because she wasn’t concentrating as well…it’s not just about getting enough energy into somebody’s it’s about maintaining that control…the two have to go hand in hand. So it’s not just about the capacity to have enough energy is about the capacity to be smart.”* (PKU Clinician) *“the research I think the perhaps the confidence…that it might not affect the Phenylalanine levels.”* (PKU Clinician)
**PSYCHOLOGICAL** **EFFECTS OF PKU**	Cathartic effects of engaging in physical activity, exercise, and sport Improved and reduced self-confidence around engaging in activity with PKU Increased and decreased anxiety around engaging in activity with PKU *(Motivation)*	***Solutions:*** *“*…*just having like psychological help available for that as well and understanding how that can feed in to exercise and how to do it safely.”* (PKU Individual) *“It’s holistic, isn’t it? Because you’re only going to exercise if you’re motivated, you’re only going to be motivated if you’ve got your levels right. If you haven’t got your levels right, you’re not going to be motivated.”* (PKU Clinician) *“making certain things accessible for people with anxiety. I think the other slightly more speedy and cost effective solution would probably be exercising in your own home using zoom.”* (PKU Clinician) *“I think if I had something a bit more concrete to follow, that would not only help me, but then also if I did want to do exercise in like a sports centre, a gym or whatever, then I could I’d feel more confident in, like going to someone else that doesn’t know about PKU and helping them to understand…I feel like with exercising, I have no idea because I currently don’t know how it’s best to feel my body with the carbs and the proteins for exercise. But if I did have a clearer map of that, then I would feel more confident.”* (PKU Individual)
**SUPPORT**	Role models within PKU Clinical Family Peers *(Opportunity and Motivation)*	***Solutions:*** *“*…*if there’s some kind of like support group or network where if someone does want to do exercise just to like compare notes and experiences and things with other people that are exercising as well to kind of build confidence and momentum.”* (Industry) *“*…*it’s making sure that the parents know the benefits of exercise that could be, you know, given to their children, particularly if they I’m not familiar with exercise at all. So, I think that’s another resource for parents that they should say early on when they’re starting, as you said, looking at cookbooks and basics.”* (Industry) *“*…*nutrition companies like the companies that manufacture the supplements and the foods…like some blogs or…like a new sort of supplement that’s like specifically meant for when you’re exercising.”* (PKU Individual) *“*…*having that knowledge of other athletes who have PKU or even just enthusiasts that have PKU and I say us as people like learning from them or learning their experiences can be motivating and for a people that is seeing, you know, a role model and how they’ve dealt with it and seen the heights that they get to is incredibly motivating for everyone else to join in, in a similar or personal field. So I think yeah, just building a community of I say PKU is who have an interest in exercise I think we’ll also just kind of get the round up a little bit.”* (PKU Individual)
**KNOWLEDGE AND** **MANAGEMENT**	Limited knowledge of PKU with physical activity, exercise, and sport Clinical awareness of PKU Public awareness of PKU Awareness in the PKU community (*Capability and Opportunity)*	***Solutions:*** *“It feels like there’s two different areas for research. One is about the benefits of physical activity and reduction of sedentary behaviour in people with PKU, the physical benefits, the mental health benefits. And the second, is the impact of moving into more elite sporting activity and what changes need to be made around that.”* (PKU Caregiver) *“*…*a resource for trainers, gyms, doctors would be really useful I think.”* (PKU Individual) *“*…*a resource for employers around the barriers that we experience.”* (PKU Individual) *“…to ensure that all hospitals and clinics have that knowledge as well, because at the moment I think just in general PKU care it’s a bit of a jackpot lottery…”* (PKU Individual) *“*…*we need a resource place where we can go to and refer to when people need help. I just think there’s a lot of expectations on dietitians on what we can do, and we try and do everything, and we try and do everything to support. But sometimes we need to be able to sign post people …”* (PKU Clinician) *“…just having guidelines on, ok, well here at this level of activity, try these different things and monitor your blood levels against that like just kind of knowing what to look for, what to test for like really simple things like if I exercise 40 min a day and I take one more supplement, what’s that doing to my blood level.”* (PKU Individual) *“*…*there might be new products that we can develop to help everyday people do the exercise that they want to do.”* (Industry) *“…having the option to have more of a tailored plan from your clinic that being say you know when you go to appointments and they ask about your diet and just your life in general, if exercise and diet within exercise was kind of talked about as a repertoire, I think that should be more normalized.”* (PKU Individual) *“I would say maybe an unmet need per se, because if you see that many people interested in, they have loads of questions, it’s certainly there and it can be explored.”* (PKU Clinician) *“…home blood monitoring would be really beneficial, especially if you’re really elite in your exercise or the field of exercise or just in general training…* (PKU Individual) *“I think it should become part of the clinical discussions with your consultant, dietician, whoever, because research has shown the mental health effects of PKU and exercise is known to help mental health, so it seemed like a no brainer why wouldn’t they? I know almost every pretty much every appointment I’ve had since turning 18, I have had the pregnancy chat every time. And I turned around and said can you just stop asking…They could ask exercise questions instead.”* (PKU Individual) *“*…*there’s possibly a missed opportunity in those clinics that do group clinics where everyone all attends at the same time…there could be an educational activity around exercise.”* (Industry) *“I think it is really it is difficult, particularly if they’re at that higher level…You could take a bit of an educated guess or idea, but I think there’s just nothing…or even any evidence-based guidance on the best approach.”* (PKU Clinician) *“I think perhaps we need to focus a little bit more on it in clinic, which we perhaps don’t hugely we do ask about it with anybody PKU or not. But it’s not something we’d I think sometimes I feel like, gosh, just putting another pressure on them…* (PKU Clinician) *“I think it’s also important to talk amongst being like more active amongst the professionals as well because some of us are maybe more comfortable or have read more or have an interest because they had more patients. But then it might be that also, if you’ve got a dietitian or whatever that is not as comfortable and you know that might in indirectly might put off a bit the patient…”* (PKU Clinician) *“And I think if we reframed it culturally within the community and doctors and clinics and all that to be, how do you excel? So how do we, like be the healthiest we can be…thinking about what you said about creatine… I think we need to have more open mindedness…and monitor it with your dietitian…be in communication with them, but to expand like what we think of as the limits for the purpose of the diet”* (PKU Individual)
**OPPORTUNITIES**	Sports available to engage in Cost Influence of socioeconomic demographic status *(Opportunity)*	***Solutions:*** *“…if they’re like if they could be added physical activity events where the children can take part like in…if there’s like regular clubs…something that will stay there for the children to stay physically active.”* (PKU Caregiver) *“…money is a is a little bit of a barrier, but it’s actually and time is a bit of a barrier, but for the kids who are deprived, it’s running more holiday weekends.”* (PKU Clinician) *“I don’t see why…the NSPKU or some of the companies couldn’t offer grants…so that you can go out and do more physical activity in after school club…”* (PKU Clinician) *“Potentially bringing in to say, the NSPKU conference, a session which starts to include sport, nutrition, whatever for the younger ages, potentially different age ranges I think that would probably start to have it more on people’s radar.”* (PKU Individual) *“*…*going somewhere like for a retreat or something, just with the adults and even it would be great to have some sort of dietitian there as well, where it’s like an active vacay…they go away, and they do physical things for like a week and it’s like training you do yoga, you do swimming and all these kinds of things. And you live together…I think a PKU version of that would be awesome.”* (PKU Individual)

## Data Availability

Data is restricted due to the fact it contains some identifying information and for ethical reasons it should remain confidential.

## Abbreviations

The following abbreviations are used in this manuscript:

COM-B	Capability, opportunity, motivation, and behaviour
CVD	Cardiovascular disease
EU	European
FG(s)	Focus group(s)
GI	Gastrointestinal
PA/E	Physical activity and exercise
PAH	Phenylalanine hydroxylase
Phe	Phenylalanine
PKU	Phenylketonuria
T2D	Type 2 diabetes
Tyr	Tyrosine

## References

[1] Deslandes A.C. (2014). Exercise and mental health: What did we learn in the last 20 years?. Front. Psychiatry.

[2] Singh B., Olds T., Curtis R., Dumuid D., Virgara R., Watson A., Szeto K., O’Connor E., Ferguson T., Eglitis E. (2023). Effectiveness of physical activity interventions for improving depression, anxiety and distress: An overview of systematic reviews. Br. J. Sports Med..

[3] Wirth A., Wabitsch M., Hauner H. (2014). The prevention and treatment of obesity. Dtsch. Ärzteblatt Int..

[4] Strope M.A., Nigh P., Carter M.I., Lin N., Jiang J., Hinton S. (2015). Physical activity–associated bone loading during adolescence and young adulthood is positively associated with adult bone mineral density in men. Am. J. Men’s Health.

[5] Reiner M., Niermann C., Jekauc D., Woll A. (2013). Long-term health benefits of physical activity–a systematic review of longitudinal studies. BMC Public Health.

[6] Dempsey C., Friedenreich C.M., Leitzmann M.F., Buman M.P., Lambert E., Willumsen J., Bull F. (2020). Global public health guidelines on physical activity and sedentary behavior for people living with chronic conditions: A call to action. J. Phys. Act. Health.

[7] Martin Ginis K.A., Ma J.K., Latimer-Cheung A.E., Rimmer J.H. (2016). A systematic review of review articles addressing factors related to physical activity participation among children and adults with physical disabilities. Health Psychol. Rev..

[8] Herazo-Beltrán Y., Pinillos Y., Vidarte J., Crissien E., Suarez D., García R. (2017). Predictors of perceived barriers to physical activity in the general adult population: A cross-sectional study. Braz. J. Phys. Ther..

[9] Hollander E.L., Proper K.I. (2018). Physical activity levels of adults with various physical disabilities. Prev. Med. Rep..

[10] Kaptein S.A., Badley E.M. (2012). Sex differences, age, arthritis, and chronic disease: Influence on physical activity behaviors. J. Phys. Act. Health.

[11] Wildekamp M., Krops L., Hegeman-Seves B., Hettinga F.J., Houdijk H., Dekker R., Hoekstra F. (2024). Physical activity barriers among adults with physical disabilities or chronic diseases during and after rehabilitation. Eur. J. Adapt. Phys. Activ..

[12] Martin Ginis K.A.M., van der Ploeg H.P., Foster C., Lai B., McBride C.B., Ng K., Pratt M., Shirazipour C.H., Smith B., Vásquez M. (2021). Participation of people living with disabilities in physical activity: A global perspective. Lancet.

[13] Durstine J.L., Painter P., Franklin B.A., Morgan D., Pitetti K.H., Roberts S.O. (2000). Physical activity for the chronically ill and disabled. Sports Med..

[14] Martin J.J. (2013). Benefits and barriers to physical activity for individuals with disabilities: A social-relational model of disability perspective. Disabil. Rehabil..

[15] van Wegberg A.M.J., MacDonald A., Ahring K., Bélanger-Quintana A., Blau N., Bosch A.M., Burlina A., Campistol J., Feillet F., Giżewska M. (2017). The complete European guidelines on phenylketonuria: Diagnosis and treatment. Orphanet J. Rare Dis..

[16] Whitehall K.B., Rose S., Clague G.E., Ahring K.K., Bilder D.A., Harding C.O., Hermida Á., Inwood A., Longo N., Maillot F. (2024). Systematic literature review of the somatic comorbidities experienced by adults with phenylketonuria. Orphanet J. Rare Dis..

[17] Schulpis K.H., Tsakiris S., Traeger-Synodinos J., Papassotiriou I. (2005). Low total antioxidant status is implicated with high 8-hydroxy-2-deoxyguanosine serum concentrations in phenylketonuria. Clin. Biochem..

[18] Sitta A., Barschak A.G., Deon M., Barden A.T., Biancini G.B., Vargas R., de Souza C.F., Netto C., Wajner M., Vargas C.R. (2009). Effect of short-and long-term exposition to high phenylalanine blood levels on oxidative damage in phenylketonuric patients. Int. J. Dev. Neurosci..

[19] Rovelli V., Dicintio A., Cazzorla C. (2024). Unmet needs in phenylketonuria: An exploratory Italian survey among patients and caregivers. Curr. Med. Res. Opin..

[20] Borghi L., Moreschi C., Toscano A., Comber P., Vegni E. (2020). The PKU & ME study: A qualitative exploration, through co-creative sessions, of attitudes and experience of the disease among adults with phenylketonuria in Italy. Mol. Genet. Metab. Rep..

[21] Anjema K., van Rijn M., Verkerk H., Burgerhof J.G., Heiner-Fokkema M.R., van Spronsen F.J. (2011). PKU: High plasma phenylalanine concentrations are associated with increased prevalence of mood swings. Mol. Genet. Metab..

[22] de Castro M.J., de Lamas C., Sánchez-Pintos P., González-Lamuño D., Couce M.L. (2020). Bone status in patients with phenylketonuria: A systematic review. Nutrients.

[23] Hanusch B., Schlegtendal A., Grasemann C., Lücke T., Sinningen K. (2025). Adults with Phenylketonuria have suboptimal bone mineral density apart from vitamin D and calcium sufficiency. Front. Endocrinol..

[24] Çıkı K., Kahraman A.B., Akar H.T., Yıldız Y., Dursun A., Tokatlı A., Coşkun T., Sivri S. (2025). High prevalence of low bone mineral density in young adults with phenylketonuria. Postgrad. Med..

[25] Palermo L., Geberhiwot T., MacDonald A., Limback E., Hall S.K., Romani C. (2017). Cognitive outcomes in early-treated adults with phenylketonuria (PKU): A comprehensive picture across domains. Neuropsychology.

[26] Jahja R., van Spronsen F.J., De Sonneville L.M., van der Meere J.J., Bosch A.M., Hollak C.E., Rubio-Gozalbo M.E., Brouwers M.C., Hofstede F.C., de Vries M.C. (2017). Long-term follow-up of cognition and mental health in adult phenylketonuria: A PKU-COBESO study. Behav. Genet..

[27] Ford S., O’Driscoll M., MacDonald A. (2018). Living with Phenylketonuria: Lessons from the PKU community. Mol. Genet. Metab. Rep..

[28] Teruya K.I., Remor E., Schwartz I.V.D. (2020). Development of an inventory to assess perceived barriers related to PKU treatment. J. Patient Rep. Outcomes.

[29] Teruya K.I., Remor E., Gabe K.M., Schwartz I.V.D. (2025). Psychological aspects, challenges to treatment and resources to support patients and caregivers of PKU patients: A scoping review. Rare.

[30] Camatta G.C., de Cássia Kanufre V., Alves M.R.A., Soares R.D.L., de Carvalho Norton R., de Aguiar M.J.B., Starling A.L.P. (2020). Body fat percentage in adolescents with phenylketonuria and associated factors. Mol. Genet. Metab. Rep..

[31] Alghamdi N., Alfheeaid H., Cochrane B., Adam S., Galloway P., Cozens A., Preston T., Malkova D., Gerasimidis K. (2021). Mechanisms of obesity in children and adults with phenylketonuria on contemporary treatment. Clin. Nutr. ESPEN.

[32] MacDonald A., Van Wegberg A.M.J., Ahring K., Beblo S., Bélanger-Quintana A., Burlina A., Campistol J., Coşkun T., Feillet F., Giżewska M. (2020). PKU dietary handbook to accompany PKU guidelines. Orphanet J. Rare Dis..

[33] (2019). UK Chief Medical Officers’ Physical Activity Guidelines. https://assets.publishing.service.gov.uk/media/5d839543ed915d52428dc134/uk-chief-medical-officers-physical-activity-guidelines.pdf.

[34] González-Lamuño D., Morencos C., Arrieta F.J., Venegas E., Vicente-Rodríguez G., Casajús J.A., Couce M.L., Aldámiz-Echevarría L. (2024). Supplementation for Performance and Health in Patients with Phenylketonuria: An Exercise-Based Approach to Improving Dietary Adherence. Nutrients.

[35] Rocha J., van Dam E., Ahring K., Almeida M., Bélanger-Quintana A., Dokoupil K., Gökmen-Özel H., Robert M., Heidenborg C., Harbage E. (2019). A series of three case reports in patients with phenylketonuria performing regular exercise: First steps in dietary adjustment. J. Pediatr. Endocrinol. Metab..

[36] Jäger R., Kerksick C.M., Campbell B.I., Cribb J., Wells S.D., Skwiat T.M., Purpura M., Ziegenfuss T.N., Ferrando A.A., Arent S.M. (2017). International society of sports nutrition position stand: Protein and exercise. J. Int. Soc. Sports Nutr..

[37] Casajús J.A., Vicente-Rodríguez G., González-Lamuño D., Melina Morencos Pinedo C. (2024). Physical Exercise and Nutrition in Phenylketonuria. https://www.nutricia.com/content/dam/sn/global/nutricia/nutricia-pictures/nutricia-pictures-brand-book/metabolics/Guia_PKU_ENG.pdf.

[38] Lidegaard L.P., Schwennesen N., Willaing I., Færch K. (2016). Barriers to and motivators for physical activity among people with Type 2 diabetes: Patients’ perspectives. Diabet. Med..

[39] Baillot A., Chenail S., Barros Polita N., Simoneau M., Libourel M., Nazon E., Riesco E., Bond D.S., Romain A.J. (2021). Physical activity motives, barriers, and preferences in people with obesity: A systematic review. PLoS ONE.

[40] McPhail S.M., Schippers M., Marshall A.L., Waite M., Kuipers P. (2014). Perceived barriers and facilitators to increasing physical activity among people with musculoskeletal disorders: A qualitative investigation to inform intervention development. Clin. Interv. Aging.

[41] Casey D., De Civita M., Dasgupta K. (2010). Understanding physical activity facilitators and barriers during and following a supervised exercise programme in Type 2 diabetes: A qualitative study. Diabet. Med..

[42] Eriksson K.F., Lindgärde F. (1991). Prevention of Type 2 (non-insulin-dependent) diabetes mellitus by diet and physical exercise. The 6-year Malmö feasibility study. Diabetologia.

[43] Dombrowski S.U., Sniehotta F.F., Johnston M., Broom I., Kulkarni U., Brown J., Murray L., Araujo-Soares V. (1991). Optimizing acceptability and feasibility of an evidence-based behavioral intervention for obese adults with obesity-related co-morbidities or additional risk factors for co-morbidities: An open-pilot intervention study in secondary care. Patient Educ. Couns..

[44] Prawiradilaga R.S., Bendtsen M., Esrup S., Jørgensen N.R., Yulianto F.A., Helge E.W. (2024). Feasibility study of Community-Based training for musculoskeletal health promotion. F1000 Res..

[45] King N., Brooks J. (2018). Thematic analysis in organisational research. The SAGE Handbook of Qualitative Business and Management Research Methods: Methods and Challenges.

[46] Kennedy-Macfoy M. (2013). ‘It’s important for the students to meet someone like you.’ How perceptions of the researcher can affect gaining access, building rapport and securing cooperation in school-based research. Int. J. Soc. Res. Methodol..

[47] Guillemin M., Heggen K. (2009). Rapport and respect: Negotiating ethical relations between researcher and participant. Med. Health Care Philos..

[48] Longhurst R., Johnston L. (2023). 10 Semi-Structured Interviews and Focus Groups. Key Methods in Geography.

[49] Hennink M.M., Kaiser B.N., Weber M.B. (2019). What influences saturation? Estimating sample sizes in focus group research. Qual. Health Res..

[50] Hennink M.M. (2013). Focus Group Discussions.

[51] Woodyatt C.R., Finneran C.A., Stephenson R. (2016). In-person versus online focus group discussions: A comparative analysis of data quality. Qual. Health Res..

[52] Hayes N. (2021). Doing Psychological Research.

[53] Michie S., Van Stralen M.M., West R. (2011). The behaviour change wheel: A new method for characterising and designing behaviour change interventions. Implement. Sci..

[54] Cane J., O’Connor D., Michie S. (2012). Validation of the theoretical domains framework for use in behaviour change and implementation research. Implement. Sci..

[55] McDonagh L.K., Saunders J.M., Cassell J., Curtis T., Bastaki H., Hartney T., Rait G. (2018). Application of the COM-B model to barriers and facilitators to chlamydia testing in general practice for young people and primary care practitioners: A systematic review. Implement. Sci..

[56] Grünert S.C., Brichta C.M., Krebs A., Clement H.W., Rauh R., Fleischhaker C., Hennighausen K., Sass J.O., Schwab K.O. (2013). Diurnal variation of phenylalanine and tyrosine concentrations in adult patients with phenylketonuria: Subcutaneous microdialysis is no adequate tool for the determination of amino acid concentrations. Nutr. J..

[57] Mazzola N., Teixeira B.C., Schirmbeck G.H., Reischak-Oliveira A., Derks T.G., van Spronsen F.J., Dutra-Filho C.S., Schwartz I.V.D. (2015). Acute exercise in treated phenylketonuria patients: Physical activity and biochemical response. Mol. Genet. Metab. Rep..

[58] MacDonald A., Davies P., Daly A., Hopkins V., Hall S.K., Asplin D., Hendriksz C., Chakrapani A. (2008). Does maternal knowledge and parent education affect blood phenylalanine control in phenylketonuria?. J. Hum. Nutr. Diet..

[59] Turner G., Quigg S., Davoren P., Basile R., McAuley S.A., Coombes J.S. (2019). Resources to guide exercise specialists managing adults with diabetes. Sports Med. Open.

[60] Ten Hoedt A.E., de Sonneville L.M., Francois B., ter Horst N.M., Janssen M.C., Rubio-Gozalbo M.E., Wijburg F.A., Hollak C.E., Bosch A.M. (2011). High phenylalanine levels directly affect mood and sustained attention in adults with phenylketonuria: A randomised, double-blind, placebo-controlled, crossover trial. J. Inherit. Metab. Dis..

[61] Posadzki P., Pieper D., Bajpai R., Makaruk H., Könsgen N., Neuhaus A.L., Semwal M. (2020). Exercise/physical activity and health outcomes: An overview of Cochrane systematic reviews. BMC Public Health.

[62] Brown C.S., Lichter-Konecki U. (2016). Phenylketonuria (PKU): A problem solved?. Mol. Genet. Metab. Rep..

[63] Jurecki E.R., Cederbaum S., Kopesky J., Perry K., Rohr F., Sanchez-Valle A., Viau K.S., Sheinin M.Y., Cohen-Pfeffer J.L. (2017). Adherence to clinic recommendations among patients with phenylketonuria in the United States. Mol. Genet. Metab..

[64] Evans S., Arbuckle C., Ashmore C., Bailey S., Blaauw G., Chaudhry W., Dale C., Daly A., Downey B., Dundas J. (2025). UK Patient Access to Low-Protein Prescription Foods in Phenylketonuria (PKU): An Uneasy Path. Nutrients.

[65] Schuck F., Malgarin F., Cararo J.H., Cardoso F., Streck E.L., Ferreira G.C. (2015). Phenylketonuria pathophysiology: On the role of metabolic alterations. Aging Dis..

[66] VinÑa J., Gomez-Cabrera M.C., Lloret A., Marquez R., Minana J.B., Pallardó F.V., Sastre J. (2000). Free radicals in exhaustive physical exercise: Mechanism of production, and protection by antioxidants. IUBMB Life.

[67] Rojas-Agurto E., Leal-Witt M.J., Arias C., Cabello J.F., Bunout D., Cornejo V. (2023). Muscle and Bone Health in Young Chilean Adults with Phenylketonuria and Different Degrees of Compliance with the Phenylalanine Restricted Diet. Nutrients.

[68] Robertson L., Adam S., Ellerton C., Ford S., Hill M., Randles G., Woodall A., Young C., MacDonald A. (2022). Dietetic management of adults with phenylketonuria (PKU) in the UK: A care consensus document. Nutrients.

[69] Tankeu A.T., Pavlidou D.C., Superti-Furga A., Gariani K., Tran C. (2023). Overweight and obesity in adult patients with phenylketonuria: A systematic review. Orphanet J. Rare Dis..

[70] Rocha J.C., MacDonald A., Trefz F. (2013). Is overweight an issue in phenylketonuria?. Mol. Genet. Metab..

[71] Riedl D., Schüßler G. (2017). The influence of doctor-patient communication on health outcomes: A systematic review. Z. Für Psychosom. Med. Und Psychother..

[72] Johnston C., Davis W.E. (2019). Motivating exercise through social media: Is a picture always worth a thousand words?. Psychol. Sport Exerc..

[73] Coto J., Pulgaron E.R., Graziano A., Bagner D.M., Villa M., Malik J.A., Delamater A.M. (2019). Parents as role models: Associations between parent and young children’s weight, dietary intake, and physical activity in a minority sample. Matern. Child Health J..

[74] Lobelo F., de Quevedo I.G. (2016). The evidence in support of physicians and health care providers as physical activity role models. Am. J. Lifestyle Med..

[75] Firman S.J., Ramachandran R., Whelan K. (2022). Knowledge, perceptions, and behaviours regarding dietary management of adults living with phenylketonuria. J. Hum. Nutr. Diet..

[76] Hall J., Morton S., Hall J., Clarke D.J., Fitzsimons C.F., English C., Forster A., Mead G.E., Lawton R. (2020). A co-production approach guided by the behaviour change wheel to develop an intervention for reducing sedentary behaviour after stroke. Pilot Feasibility Stud..

[77] Heath G.W., Parra D.C., Sarmiento O.L., Andersen L.B., Owen N., Goenka S., Montes F., Brownson R.C. (2012). Evidence-based intervention in physical activity: Lessons from around the world. Lancet.

[78] Ilgaz F., Ford S., O’Driscoll M.F., MacDonald A. (2023). Adult PKU Clinics in the UK—Users’ Experiences and Perspectives. Nutrients.

[79] Jones H., Crozier A., George K., Miller G., Whyte G.P., Rycroft J., Scott A., Buckley J.P., McGregor G., Askew C.D. (2024). Establishment of clinical exercise physiology as a regulated healthcare profession in the UK: A progress report. BMJ Open Sport Exerc. Med..

[80] Daly A., Evans S., Pinto A., Ashmore C., MacDonald A. (2021). Protein substitutes in PKU; their historical evolution. Nutrients.

[81] Matthews A., Jones N., Thomas A., van den Berg P., Foster C. (2017). An education programme influencing health professionals to recommend exercise to their type 2 diabetes patients–understanding the processes: A case study from Oxfordshire, UK. BMC Health Serv. Res..

[82] Halabchi F., Alizadeh Z., Sahraian M.A., Abolhasani M. (2017). Exercise prescription for patients with multiple sclerosis; potential benefits and practical recommendations. BMC Neurol..

[83] Del Giacco S.R., Firinu D., Bjermer L., Carlsen K.H. (2015). Exercise and asthma: An overview. Eur. Clin. Respir. J..

[84] Gloeckl R., Marinov B., Pitta F. (2013). Practical recommendations for exercise training in patients with COPD. Eur. Respir. Rev..

[85] Zhang C., Wei S., Zhao Y., Tian L. (2023). Gender differences in research topic and method selection in library and information science: Perspectives from three top journals. Libr. Inf. Sci. Res..

[86] Schneider S.J., Kerwin J., Frechtling J., Vivari B.A. (2002). Characteristics of the discussion in online and face-to-face focus groups. Soc. Sci. Comput. Rev..

